# Mango Consumption Is Associated with Increased Insulin Sensitivity in Participants with Overweight/Obesity and Chronic Low-Grade Inflammation

**DOI:** 10.3390/nu17030490

**Published:** 2025-01-29

**Authors:** Katherine D Pett, Peter Geevarghese Alex, Casey Weisfuss, Amandeep Sandhu, Britt Burton-Freeman, Indika Edirisinghe

**Affiliations:** Center for Nutrition Research, Department of Food Science and Nutrition, Institute for Food Safety and Health, Illinois Institute of Technology, Chicago, IL 60616, USA; katherine.d.pett@gmail.com (K.D.P.); pgeevarghesealex@iit.edu (P.G.A.); cweisfuss@iit.edu (C.W.); asandhu2@iit.edu (A.S.); bburton@iit.edu (B.B.-F.)

**Keywords:** glucose, glucoregulatory, HOMA-IR, Matsuda index, insulinogenic index, Nrf2, inflammation

## Abstract

**Background/Objectives:** Chronic low-grade inflammation is associated with insulin resistance and poor glycemic control, leading to the development of type 2 diabetes mellitus (T2DM). The present study investigated the effect of regular mango intake on inflammation and insulin sensitivity in participants with overweight or obesity and chronic low-grade inflammation. **Methods:** A human clinical study was performed using a randomized, controlled, two-arm, parallel design with a 2 h oral glucose tolerance test (OGTT) administered before and after 4 weeks (4 W) of mango or control product intake (1 cup/twice a day). Fasting and time course blood sampling for 2 h post-OGTT were analyzed for effects on plasma metabolic and inflammation endpoints using analysis of covariance and repeated-measure approaches (SAS 9.4). **Results:** Forty-eight adults (37.6 ± 2.8 years, 30.5 ± 4.1 BMI kg/m^2^) completed the study. Markers of inflammation (IL-6, TNFα, hs-CRP) were not different at the end of 4 W (*p* > 0.05). The intervention did not significantly influence fasting glucose concentrations; however, insulin was significantly lowered with the mango compared to the control intervention (8.2 ± 1.2 vs. 15.3 ± 1.2 µIU/mL respectively, *p* = 0.05). Furthermore, the Homeostasis Model Assessment of Insulin Resistance (HOMA-IR), along with the disposition index (DI), was significantly improved in the mango compared to the control interventions (HOMA-IR, 2.28 ± 1.19 vs. 4.67 ± 1.21, *p* = 0.03; DI, 2.76 ± 1.02 vs. 5.37 ± 1.03, *p* = 0.04). Mean insulin concentrations were also significantly lower at W4 compared to W0 after the OGTT in the mango vs. control intervention (intervention × week effect, *p* = 0.04). Relative expression of nuclear factor erythroid 2-related factor 2 (Nrf-2), a gene regulating endogenous antioxidant defense, was non-significantly increased twofold in the mango intervention (W4 vs. W0). **Conclusions:** Collectively, the data suggest that mango intake increased insulin sensitivity in individuals with chronic low-grade inflammation, possibly through activating Nrf-2 genes and increasing cellular antioxidant status. The data warrant further research on consuming mango fruit as part of a dietary pattern to address insulin resistance and the mechanisms underpinning the actions of mango intake.

## 1. Introduction

Chronic low-grade inflammation is a significant contributor to metabolic syndrome and the outcomes of its complications, including heart disease, diabetes, and cognitive decline [1,2,3]. In particular, chronic low-grade inflammation has been shown to influence the development of type 2 diabetes mellitus (T2DM), mediating insulin resistance and poor glycemic control [4,5,6]. C-reactive protein (CRP) is the standard clinical marker for chronic low-grade inflammation and is an independent risk factor for cardiovascular disease (CVD) [7]. The prevalence of T2DM and CVD is higher in obesity, which is a pro-inflammatory state [8,9]. The global prevalence of overweight and obesity is 12.5%, with 6.3% of the population having T2DM and 9.1% having prediabetes (defined as fasting glucose >100 mg/dL) [10]. Weight loss is the first line of therapy, having substantial metabolic and anti-inflammatory effects with as little as 5–10% weight loss [11]. Dietary interventions that target biological mechanisms of metabolic dysregulation, such as alleviating chronic low-grade inflammation, could play an important role in tackling the metabolic disease burden even when weight loss is not achievable [12]. The global burden of disease research has identified major dietary risks in disability-adjusted life years due to CVD and T2DM, and low fruit intake is among the top three contributors [13].

Mango is a tropical fruit with a unique composition consisting of dietary fiber, essential vitamins and minerals, and various nutrient-like bioactive compounds [14]. NHANES data (2001–2018) indicate that mango consumers have a higher diet quality than non-consumers, and male and adolescent consumers have lower body weight, smaller waist circumference, and lower body mass index (BMI) z-scores, respectively, than non-mango consumers [15]. Additionally, several groups have reported lower postprandial glycemic responses after consuming mango in various human intervention studies [16,17,18,19,20]. The observed acute effects could be due to the unique nutrient composition of mangoes, including fibers and other nutrient-like bioactive compounds. Fibers have known glycemia-modulating effects, and extracted compounds from mango, including mangiferin, have shown hypoglycemic action in rat animal models [21]. Research on the role of mango and its components in inflammation is slowly progressing. Data from animal studies show improved inflammatory status after mango interventions, with reports of reduced pro-inflammatory markers [22,23,24,25] or increased anti-inflammatory markers [25]. However, results from human studies are mixed, showing a reduction in select inflammatory markers [16,17,20,26] and no effect in other studies [17,26]. The discrepancies observed could be due to study models, populations, feeding duration, and doses used in different studies.

Feeding studies in humans suggest that mango intake contributes to glucose control, although the mechanisms underlying the effects are not clear [16,20,27]. One possibility is through reducing systemic inflammation, as suggested by the results of a responder (*n* = 8)/non-responder (*n* = 18) analysis after 8-week intake of mango pulp and results from a 12-week randomized controlled trial (*n* = 27) in individuals with overweight/obesity, both reporting reduced CRP and decreased glucose after mango intake [16,27]. Decreased HOMA-IR along with changes in gene expression were also reported in the responder group analysis, suggesting glucose control may be achieved through improvements in insulin sensitivity [27]. However, there are limited data from controlled chronic feeding studies with mango, and none have assessed peripheral insulin sensitivity. Therefore, we tested the hypothesis that regular mango intake will improve inflammation status in people with overweight/obesity (OW/OB) and chronic low-grade inflammation, resulting in increased insulin sensitivity.

## 2. Materials and Methods

### 2.1. Ethics, Participants, and Study Design

The study was conducted in accordance with the Declaration of Helsinki and approved by the Institutional Review Board of the Illinois Institute of Technology (protocol IRB_2020_57, approval date 2 March 2020). Informed consent was obtained from all participants involved in the study. All procedures were performed at the Clinical Nutrition Research Center (CNRC), Illinois Institute of Technology, Chicago, IL 60616. The study was registered at ClinicalTrials.gov (NCT04726293).

Potential participants were screened for inclusion criteria: BMI ≥ 25 kg/m^2^, high-sensitivity C-reactive protein (hs-CRP) >1.0 and <10.0 ng/L (as per cut points established by the Centers for Disease Control and Prevention (CDC)/American Heart Association (AHA) for chronic low-grade inflammation), fasting blood sugar >100 mg/dL and <126 mg/dL [28], and age 20–60 years. Exclusion criteria included regular use of multivitamins or supplements high in polyphenolic compounds, statins, vegan dietary patterns, and clinical diagnoses of chronic disease related to metabolic illness. Participants were recruited from the greater Chicagoland area from June 2020 to December 2023.

**Figure 1:**
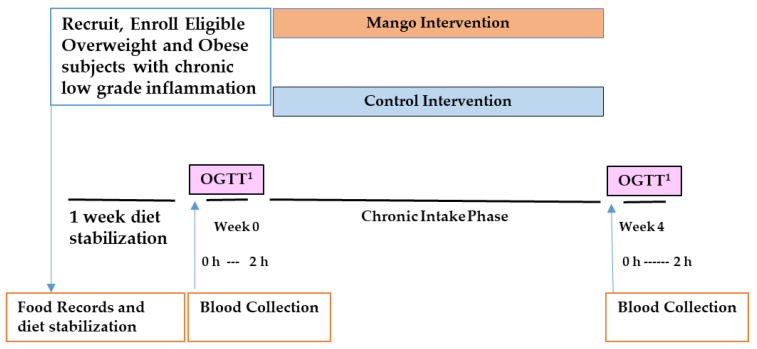
Study schema. Randomized, single-blind, parallel design for 4-week study intervention. ^1^ OGTT: oral glucose tolerance test. 0 h–2 h: Blood samples were collected at 0, 30, 60, 90, and 120 min.

The study was a 4-week randomized, placebo-controlled, single-blinded, parallel-design clinical trial. Participants visited the CNRC five times for assessments and study product pickup (Figure 1). The two main assessment days were at the beginning of week 0 and at the end of week 4, which were identical and included an oral glucose tolerance test (OGTT) procedure. Starting a week before their first OGTT, participants were asked (and provided guidance) to follow their usual diet with the exception of mangoes and other high-(poly)phenol foods. Participants continued this practice throughout the study. The night before each OGTT, participants were instructed to consume a consistent dinner meal reimbursed by the clinical staff. Three-day food records were collected before each study day to monitor compliance with OGTT visit day dietary preparation protocols. At each study day visit, anthropometrics, body composition, and vital signs were measured. Gastrointestinal tolerability of the interventions was assessed through an online questionnaire, including questions about nausea, bloating, gas/flatulence, bowel consistency, and abdominal pain, administered to participants between each study day visit.

### 2.2. Oral Glucose Tolerance Test (OGTT)

At the beginning of week 0 and at the end of week 4, OGTTs were performed, using 75 g dextrose in 150 mL of water. Participants were advised to consume the glucose drink in 5 min. Venous blood collection was through an indwelling catheter placed at the antecubital fossa vein in the arm. Sampling occurred before the glucose drink (Time 0), and additional samples were taken at 30, 60, 90, and 120 min intervals after the start of the glucose drink. Blood was collected in EDTA tubes, placed on ice immediately, and centrifuged at 4 °C and 453× *g* for 10 min within 30 min, after which plasma was separated and stored at −80 °C until analysis. Before administering the glucose drink, fasting venous blood was collected in PAXgene^®^ (BD Bioscience, Franklin Lakes, NJ, USA) tubes for RNA extraction and gene expression analysis.

### 2.3. Study Interventions

Mangoes (Kent and Keitt varieties) were purchased from Costco Wholesale and Amazon Fresh stores around the Chicagoland area. Fresh-ripened mangoes were peeled, cut into small pieces for blending, and stored in a −20 °C freezer. All food processing procedures were performed in the metabolic kitchen following appropriate food safety procedures. Due to the limited availability of mangoes and freezer space, fresh mangoes were purchased and processed five times during the study (within two years).

Energy-matched (100 kcal) mango and control interventions were prepared using the recipe given in Table 1. One serving of the mango intervention consisted of 126.9 g of Kent mango and 38.1 g of Keitt mango. The base of the control intervention was a commercially available Italian ice. Each participant was given a one-week supply (+2 days extra) of mango or control products, with two-week supplies provided as needed for participants with difficulty maintaining weekly pickup visits. The mango and control products were freshly prepared and frozen at −20 °C in 5.5-ounce cups with lids one day before pickup. Participants were given mango or control products in a cooler bag and advised to keep them in the freezer and take two out per day in the refrigerator to soften the day before consumption. They were asked to consume 2 cups/day (1 in the morning and 1 in the afternoon). Participants were given a diary to record the day and time of consumption to monitor compliance.

### 2.4. Study Endpoint Analyses

#### 2.4.1. Glucose, Insulin, hs-CRP, and Lipid Profile

Glucose, insulin, hs-CRP, and lipid profiles were measured from blood samples collected in EDTA-coated vacutainer tubes. After collection, the tubes were placed on ice and centrifuged for 15 min at 4 °C and 453× *g* within 30 min of collection. The plasma was then aliquoted and stored at −80 °C until analysis. These assays were assessed using the Randox Daytona automated clinical analyzer (Randox Laboratories) and were determined according to the manufacturer’s instructions, with appropriate quality controls.

#### 2.4.2. IL-6 and TNF-α

ELISA assays were used to measure interleukin 6 (IL-6) and tumor necrosis factor alpha (TNFα) concentrations in plasma samples. The ELISA test was conducted using a Quantikine^®^ (R & D Systems, Minneapolis, MN, USA) HS ELISA human IL-6 immunoassay kit (R & D, catalogue number HS600C) and a Quantikine^®^ HS ELISA human TNFα immunoassay kit (R & D, catalogue number SSTA00E). In brief, frozen samples (−80 °C) were thawed in the morning, and the assay was performed according to the manufacturer’s instructions. To avoid variations, the samples belonging to the same subject were assayed in the same ELISA plate. Unknown sample concentrations were calculated using 7-point standard curves (pg/mL). Quality control samples were used to calculate intra/inter-assay variability. Those sample concentrations above the standard curve (10 pg/mL) were re-assayed using appropriate dilutions.

#### 2.4.3. RNA Extraction and q-PCR Procedure

For the RNA extraction procedure, blood samples were collected from participants at the fasting pre-OGTT timepoint and placed into PAXgene blood tubes. The tubes were incubated for 2 h at room temperature to stabilize RNA. After incubation, the tubes were transferred to a −20 °C freezer overnight. The next day, the samples were moved to a −80 °C freezer for long-term storage until RNA extraction.

RNA was isolated from the stored PAXgene-processed blood samples using a Thermo Scientific GeneJET stabilized and fresh whole blood RNA kit (REF—K0871) following the manufacturer’s instructions. Briefly, RNA was eluted in RNase-free water, and its concentration was measured using a Qubit 4 fluorometer (Invitrogen^TM^ by Thermo Fisher Scientific, Waltham, MA, USA—Pub No-MAN0017210). The extracted RNA was stored at −80 °C until further use.

Complementary DNA (cDNA) was synthesized from the total RNA using an Applied Biosystems^TM^ High-Capacity RNA-to-cDNA^TM^ Kit (cat-4387406, lot-2905856). The kit was stored at −25 °C to −15 °C before use. Reverse transcription (RT) buffer (2X) mix and RT enzyme mix reagents (20X) were used for reverse transcription following the manufacturer’s instructions. The RT reaction was performed using a QuantStudio^®^ 3 system (Waltham, MA, USA, pub no-100025904). cDNA was stored in a −20 °C freezer.

Stored cDNA was used for quantitative real-time polymerase chain reaction (qPCR) using the PowerUp^TM^ SYBR^TM^ Green Master Mixture (Applied Biosystems, Waltham, MA, USA, CAT-A25741, LOT-2755313). Each reaction contained cDNA (1–10 ng), PowerUp^TM^ SYBR^TM^ Green Master Mix, forward and reverse primers, and nuclease-free water. All the primers were ordered from Integrated DNA Technologies. Toll-like receptor 2 (TLR2) gene (ref seq number: NM_003264) sequence: 5′-CCATTGCTCTTTCACTGCTTTC-3′/5′-ATGACCCCCAAGACCCA-3′. Toll-like receptor 4 (TLR4) gene (ref seq number: NM_138557) sequences: 5′-GAGTATACACATTGCTGTTTCCTGTTG-3′/5′-ACCCCATTAATTCCAGACACA-3′. Nuclear factor erythroid 2-related factor 2 (Nrf2) (ref seq number NM_001145412) sequences: 5′-CGTAGCCGAAGAAACCTCAT-3′/5′-ACATCCAGTCAGAAACCAGTG-3′. The housekeeping gene used for normalization was GAPDH (ref seq number: NM_002046), with the primer sequence 5′-ACATCGCTCAGACACCAT-3′/5′-TGTAGTTGAGTTCAATGAAGGG-3′. Threshold cycle (Ct) values were obtained for each gene, including the housekeeping gene. The relative expression of target genes was calculated and compared across all samples. Gene expression levels were quantified using the 2^−∆∆Ct^ method, where Ct values were normalized against the glyceraldehyde-3-phosphate dehydrogenase (GAPDH) reference gene. Gene expression at baseline (before any intervention) was used as the base to calculate the fold increase.

### 2.5. Data Analysis

#### Calculations and Statistical Analysis

Several equations were used to assess glucoregulation. The Homeostasis Model Assessment of Insulin Resistance (HOMA-IR) equation estimates hepatic insulin resistance using fasting plasma glucose and insulin concentrations [29]. The Matsuda index estimates whole-body insulin sensitivity and was calculated using fasting plasma glucose, fasting plasma insulin, and glucose and insulin concentrations after the OGTT [30,31]. The disposition index measures how well beta cells compensate for insulin resistance and is calculated by multiplying the Matsuda index by the insulinogenic index [30]. The area under the curve (AUC) estimates the relative systemic exposure of an analyte and was calculated for insulin and glucose concentrations measured at 0, 30, 60, 90, and 120 min (AUC_0–120min_) using the trapezoidal rule [32]. The equations are given below:

HOMA-IR = (fasting insulin × fasting glucose)/22.5

Matsuda Index = 10,000/square root [(fasting glucose × fasting insulin) × (mean glucose × mean insulin)]

Insulinogenic Index = (I30−I0)/(G30−G0)

where I = insulin and G = glucose concentrations at the given time point (30 min and 0 min).

Disposition Index = Matsuda Index × Insulinogenic Index

AUC = (C1 + C2)/2 × T2−T1 (C = Concentration, T = Time)

Statistical analyses, randomization schedules, and sample size estimates were performed using SAS version 9.4 (SAS Institute Inc., Cary, NC, USA). Demographic and baseline clinical variables were tabulated from descriptive statistics and tested for differences between intervention groups as appropriate. Shapiro–Wilks test histograms and PP and QQ plots were used to assess the normality distributions of all outcome variables. Non-normal variables were log_10_-transformed and retested before statistical analyses. Outlier analysis was determined from box plot review and removed as part of a sensitivity analysis if greater than 1.5 the interquartile range. Fasting variables, including glucose, insulin, glucoregulatory indicators (HOMA-IR, insulinogenic index, Matsuda index, disposition index), inflammatory markers, and glycemic AUCs were tested for differences between the interventions at week 4 by analysis of covariance (ANCOVA), with baseline values as the primary covariate in the model. Sex, BMI (kg/m^2^), and other covariates were included as appropriate after testing for significance. Postprandial glucose and insulin time–concentration curves after the OGTT were analyzed using repeated-measure analysis of variance (RM-ANOVA), with intervention, time, and their interaction as the main factors. Gene expression data were analyzed using an unpaired *t*-test.

Results of the statistical analyses are presented as least square means (LSMs) ± standard error of mean (SEM) unless indicated otherwise. Statistical significance was based on a 2-sided comparison at the 5% significance level under a null hypothesis of no difference between interventions. Multiple comparisons were corrected using Tukey–Kramer in mixed-model procedures. Statistical significance was determined at *p* < 0.05.

Randomization schedules and sample sizes were generated using PROC PLAN. The sample size of 44 participants was estimated based on data from our previous work, which showed >80% power for detecting significant differences between interventions for inflammatory marker endpoints [33,34].

## 3. Results

### 3.1. Participant Demographics and Characteristics

**Figure 2:**
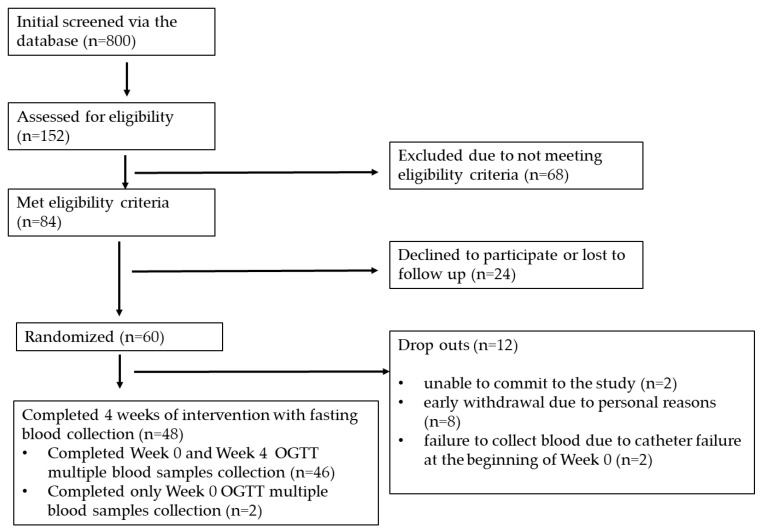
Consolidated Standards of Reporting Trials (CONSORT) diagrams for study recruitment.

A total of 152 participants were assessed for eligibility, of which 84 met the inclusion criteria. Sixty participants were randomized to the mango or control interventions based on a computer-generated randomization code. Overall, 12 participants dropped out at various stages, and 48 completed the study interventions. Forty-six participants completed the OGTT procedure before and after 4 weeks of intervention, and two participants could not complete the OGTT procedure due to catheter failure during one of their visits (Figure 2). Demographic and baseline metabolic, inflammatory, and other health indices are given in Table 2.

### 3.2. Inflammatory Markers Assessment in Response to Mango or Control Intervention

**Figure 3:**
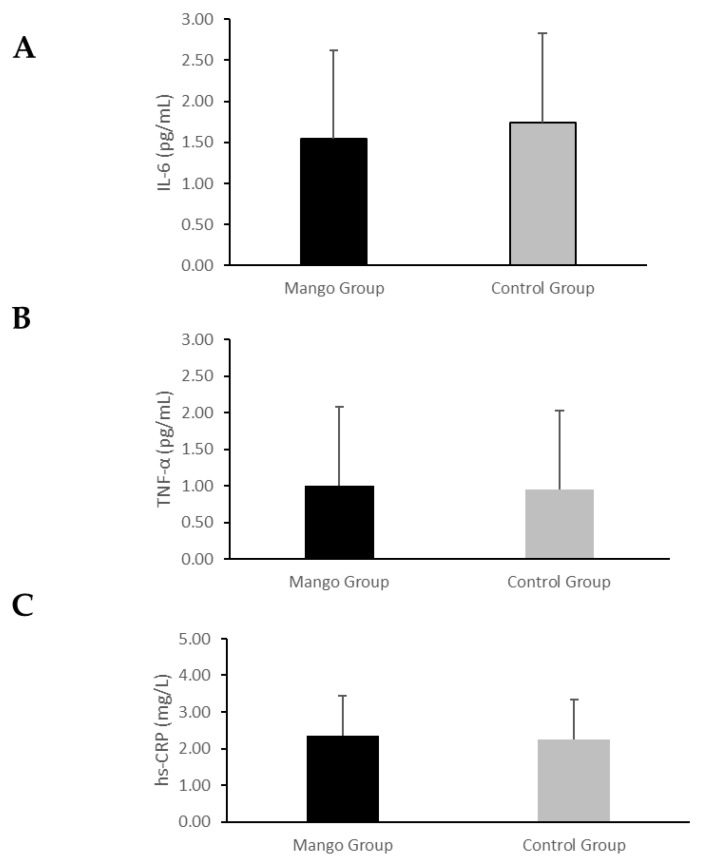
Effect of 4 weeks of mango and control interventions on fasting inflammatory markers in people with chronic low-grade inflammation. (**A**) = IL-6, (**B**) = TNFα, and (**C**) = hs-CRP. Data are presented as least squares means (LSMs) ± SEM, *n* = 46, derived from ANCOVA by PROC MIXED in SAS 9.4, with the main effects of the intervention at week 4 and baseline values as the covariate. No differences between interventions were observed for any of the variables.

The effect of four weeks of mango or control interventions on chronic low-grade inflammation was assessed using PROC MIXED (ANCOVA) methods as described in the statistical section. The results of the analysis are shown in Figure 3. No significant effect of the interventions was apparent for any inflammation variables, *p* > 0.05. All assays had intra and inter-assay CVs less than 10%, as recommended by the manufacturer.

### 3.3. Body Weight Response to Mango and Control Interventions

Changes (week 0–week 4) in body weight were not different after the mango intervention, but were different after the control intervention (mango 0.27 ± 0.30 kg vs. control intervention 0.87 ± 0.21 kg), resulting in a statistically significant difference in the changes between diets (*p* < 0.001) (Table 3).

### 3.4. OGTT Response to Mango and Control Interventions

**Figure 4:**
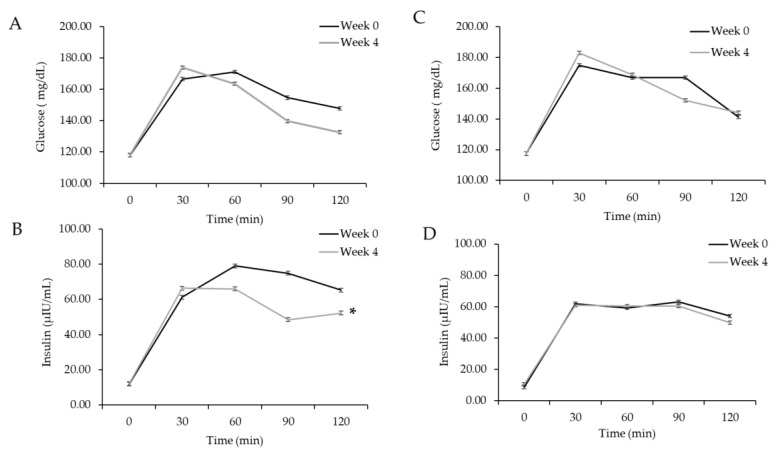
Glucose and insulin time by concentration curves following an oral glucose tolerance test (OGTT). (**A**,**B**) OGTT results before and after 4 weeks of the mango intervention. (**C**,**D**) Data from OGTT results before and after the control intervention. Data are presented as least squares means (LSMs) ± SEM, *n* = 46. Data were analyzed by RM-ANOVA via PROC MIXED in SAS, with the main effects of intervention, time, week, and their interactions. No significant interactions were observed for glucose. * A significant intervention-by-week interaction was apparent for insulin, indicating reduced insulin after OGTT at week 4 compared to week 0 in the mango intervention (*p* = 0.04), but not the control intervention (*p* = 0.99).

OGTT-mediated glucose and insulin concentrations after 4 weeks of mango and control interventions in participants with chronic low-grade inflammation were assessed using RM ANOVA, as described in the statistics section. Glucose after the OGTT was not influenced by intervention; however, insulin concentrations in response to the OGTT were significantly lower after 4 weeks of mango intervention compared to the group’s week 0 responses (Figure 4A,B, *p* = 0.04). Insulin concentrations did not differ between week 0 and week 4 after the control intervention (Figure 4C,D *p* = 0.99).

### 3.5. Fasting Glucose, Insulin, and Glucoregulatory Indices Assessments in Response to Mango and Control Interventions

At the end of week 4, fasting glucose concentrations were not significantly different between mango and control interventions (119.67 ± 1.02 mg/dL vs. 116.95 ± 1.02 mg/dL, *p* = 0.51). However, fasting insulin concentrations at the end of week 4 were significantly lower in the mango intervention compared to the control intervention (8.2 ± 1.16 µIU/mL vs. 15.26 ± 1.18 µIU/mL, *p* = 0.05). This difference in insulin concentrations at week 4 was consistent, with a significant difference between interventions in the changes from week 0 to week 4 (delta) (*p* = 0.05). Additionally, HOMA-IR was significantly lower after the mango intervention than the control intervention (2.28 ± 1.19 vs. 4.67 ± 1.21, *p* = 0.03), as were the delta differences between interventions (*p* = 0.0009). Age was a significant covariate in the model for insulin and HOMA-IR (*p* = 0.02 and *p* = 0.05, respectively), but no interaction with the intervention was observed. Trends toward improvement in the Matsuda index and the insulinogenic index were observed in the mango intervention group compared to the control group, although they were not significant (*p* = 0.07 and 0.09, respectively). The disposition index was significantly improved in the mango intervention group compared to the control group (*p* = 0.04), and the effect was influenced by BMI (*p* = 0.04). Data are presented in Table 4.

### 3.6. Lipid Profile Assessments in Response to Mango and Control Interventions

Lipids were not influenced by dietary interventions (Table 4). No significant differences between interventions in fasting total cholesterol, LDL cholesterol, HDL cholesterol, or triglycerides were observed (*p* > 0.05)

### 3.7. Gene Expression: Cellular Defense, and Inflammation in Peripheral Blood Mononuclear Cells (PBMCs)

**Figure 5:**
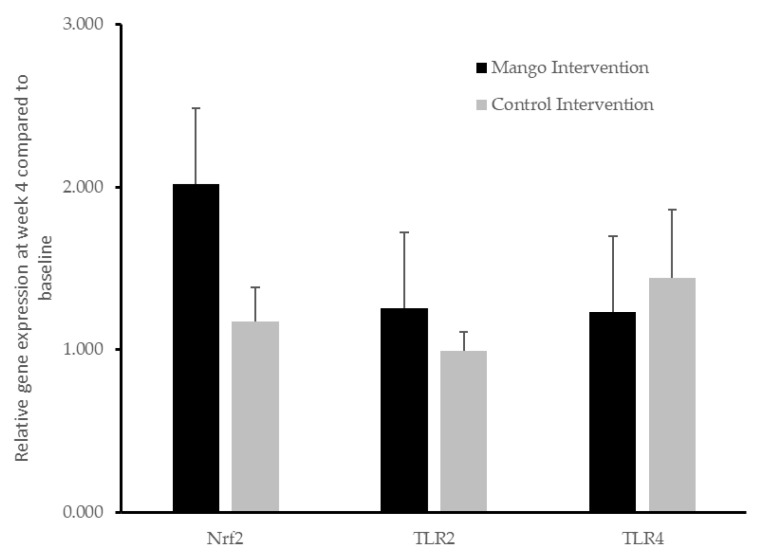
Relative gene expression at week 4 compared to baseline levels after providing participants with mango or control interventions. Data are presented as means ± SEM of relative gene expression, *n* = 46. No effect of intervention was observed for nuclear factor erythroid 2-related factor 2 (Nrf2) or Toll-like receptor 2 and 4 (TLR2 and TLR4) relative gene expression, *p* > 0.05.

Relative gene expression in PBMCs in participants who consumed mango or control interventions was assessed by the 2^−∆∆Ct^ method using qPCR. Relative expression of nuclear factor erythroid 2-related factor 2 (Nrf2) and Toll-like receptor 2 and 4 (TLR2 and TLR4) genes did not differ between interventions (*p* > 0.05). A non-significant increase (around double) in mean Nrf2 gene expression was observed at the end of the mango intervention compared to baseline gene expression levels (Figure 5).

## 4. Discussion

The primary working hypothesis of this project was that regular mango intake would reduce inflammation in individuals with chronic low-grade inflammation, resulting in increased insulin sensitivity. This hypothesis stems from data indicating that inflammation is a mechanism that contributes to insulin resistance and data in preclinical and clinical models that suggest that mango or its components have anti-inflammatory activity [22,23,26]. The results indicated that the 4-week mango intervention did not significantly affect inflammatory indices measured by IL-6, TNFα, and hs-CRP. However, fasting insulin concentrations were significantly lowered with the mango intervention compared to the control intervention, and insulin resistance status was improved, evidenced by changes in HOMA-IR and disposition index. These data indicate that mango intake daily for 4 weeks increased insulin sensitivity, reducing the amount of insulin required to maintain glucose in people with chronic low-grade inflammation, but the effect was not through an inflammation pathway based on the markers measured in this study. Alternatively, the effects may be related to a change in cellular redox activity deserving follow-up research.

Insulin resistance is a metabolic condition where tissues are resistant to the action of insulin, requiring higher-than-normal insulin concentrations to manage glucose. Insulin resistance may exist at hepatic or peripheral tissue (i.e., muscle, fat) levels or both. In normal physiology, hepatic insulin resistance manifests in uncontrolled glucose production by the liver in fasting conditions. Peripheral insulin resistance is impaired glucose uptake from blood into extrahepatic tissues in postabsorptive and fasting states [35,36]. The present study used the HOMA-IR equation, which relies on fasting glucose and insulin values to estimate hepatic insulin resistance, and the Matsuda index, which uses data from an OGTT to estimate whole-body insulin sensitivity. The Matsuda index considers the metabolic clearance rate during the OGTT [30,31]. The HOMA-IR results indicated reduced insulin resistance, even though fasting glucose was not reduced. Instead, it appears less insulin was required to manage hepatic glucose production. Perhaps we would have found concomitantly reduced fasting glucose with a longer-duration study. Rosas et al. (2022) reported decreased fasting glucose after 12 weeks of mango consumption compared to a cookie control in people with overweight or obesity (*n* = 27) [16]. We also found that the Matsuda index was higher (higher values = better insulin sensitivity) after the mango intervention compared to the control intervention, although the difference was not significant (*p* = 0.07). The insulinogenic index, which measures how well a person’s beta cells respond to a glucose load, also showed improvement in the mango group compared to the control group (*p* = 0.09), also not significant, but the DI was significantly different in the mango group, indicating beta cells compensating for insulin resistance (*p* = 0.04). Overall, the glucoregulatory indices associated with insulin sensitivity point toward a beneficial effect of mango intake at the level of the pancreas and the liver (and possibly peripherally), resulting in reduced insulin resistance.

The available literature on acute and chronic feeding of mango or mango-associated component consumption supports reduced postprandial glycemia and improved insulin activity. A study conducted by Pinneio et al., 2022 with fresh mangoes or isocaloric low-fat cookies (100 kcal) showed significantly lower postprandial insulin at 45 min after mango intake compared to intake of low-fat cookies (*p* ≤ 0.05) [19]. Glucose was also significantly lower after mango compared to low-fat cookie intake at 30 min (*p* ≤ 0.05) [19]. In another crossover study design (*n* = 34 healthy weight) postprandial glucose concentrations were significantly reduced after consuming fresh or dried mango compared to white bread (*p* < 0.05) [37]. A 6-week clinical study (mango flesh, 400 g/day) in participants with obesity resulted in reduced HbA1c (*p* = 0.006), despite no changes in fasting insulin or glucose [20]. Interestingly, a study by Keathley et al., 2022 in overweight and obese participants (*n* = 27) showed that 280 g/day of mango pulp consumption for eight weeks reduced 2 h plasma glucose concentration after a 75 g oral glucose tolerance test (10.5% reduction/−0.58 ± 1.03 mmol/L, *p* = 0.008, *n* = 27) [18]. These data support the actions of mango in glucoregulation during the postprandial state. The results from the present study suggest the effects may be due to improved insulin sensitivity.

Mango is a tropical fruit with various nutrients and nutrient-like bioactive components that have beneficial health effects. The sweetness of mango has raised concerns about sugar content and weight gain in obesity and diabetes, which has not been supported by science [14]. The present study monitored body weight and found no change in weight after the mango diet, although a modest but significant weight gain of ~1 kg was observed after the control diet. The difference in weight change was significantly different between diets. The data from the present study support previous findings that people do not gain weight with mango consumption [16,19,27,38]. One cup equivalent of mango (~165 g, 100 kcal) provides 100% of the daily value of vitamin C, 35% of the daily value of vitamin A, and 12% of the daily value of fiber [14]. Furthermore, mangoes are a rich source of bioactive polyphenols [14]. Vitamins A and C and polyphenols are known for their antioxidant activity.

The present study was based on the hypothesis that mango intervention is associated with improved insulin sensitivity via a mechanism associated with reduced inflammation. However, our data did not support an association with inflammation. One possibility is that the study was performed during the COVID-19 pandemic. Although the screening procedures excluded participants who had recent COVID-19 illness, symptoms, or vaccination within the week before the study began, it is still possible that the inflammatory effects of prior infection or vaccination could have influenced the results. [39]. In the present study, the SEM values of IL-6 were very high compared to the studies we used for power calculations [33]. The study also measured TL2, TL4, and Nrf-2 gene expression levels in PBMCs before and after the interventions. It is known that nuclear factor-kappa B transcription factor (NFкB)-associated inflammatory signaling is mediated through TLRs [40], and relative gene expression compared to their baseline (before the intervention) was not evident in the present study. However, relative Nrf-2 gene expression doubled in the mango group (although it was not significant). It is possible that the Nrf-2 gene expression did not reach a significant value as the study was not powered based on Nrf-2 gene expression. Nevertheless, these data are interesting, as Nrf-2 is a transcription factor that regulates genes involved in cellular defense against oxidative stress [41]. Mango phytonutrients may have improved the antioxidant status of our participants with chronic low-grade inflammation, as inflammation typically coincides with redox imbalances and oxidative stress. Relevant to the gene expression data in the present study, a single-arm clinical trial conducted by Keathley et al., 2022, used changes in gene expression after mango intake (pulp 280 g/day) for 8 weeks to identify responders (*n* = 8) and non-responders (*n* = 18) to the intervention. The authors observed several catabolic and metabolic processes, and oxidative, immune, and inflammatory biological processes were associated with gene expressions in responders. Significant changes in the quantitative insulin sensitivity check index (QUICKI) and HOMA-IR were reported in responders vs. non-responders [27]. Rosas et al., (2022) reported significantly decreased glucose, hs-CRP, and aspartate transaminase activity in a 12-week randomized crossover trial in overweight individuals consuming fresh mango (100 kcal/day) and cookies separated by a 4-week washout period. The study also reported total antioxidant capacity (TAC) significantly increased following mango consumption (*p* < 0.05) following 12 weeks of intake [16]. Collectively, these data suggest that regular mango consumption, associated with improved glucoregulation and insulin sensitivity, may be mediated by a mechanism related to improved antioxidant status.

A strength of this study was its design: a 4-week randomized, placebo-controlled, single-blinded, parallel design conducted in free-living conditions, alongside assessments of both fasting and postprandial glycemic responses. This approach enabled a comprehensive evaluation of hepatic and peripheral insulin sensitivity. However, the study also had limitations. The study was conducted during the COVID-19 pandemic, which may have impacted the inflammation data and interpretation of results (higher variance than we typically see in the markers measured). Viral infections and vaccinations increase systemic inflammation, which were beyond our control. While we measured selected genes for expression and inflammatory markers, a more comprehensive biomarker panel, including specific proteins, could have yielded insights not apparent in the current study. Additionally, the absence of mango metabolites is a concern, and we intend to measure these in future research. While the study has some limitations in fully elucidating the potential inflammatory mechanisms related to insulin sensitivity, the strength of the data presented remains important.

## 5. Conclusions

In summary, the present study provides evidence that mango fruit intake plays a role in insulin sensitivity, as demonstrated by lowered fasting insulin concentrations and HOMA-IR and improved DI after 4 weeks of daily mango intake in individuals with chronic low-grade inflammation. The data do not support changes in inflammation status as the mechanism underpinning the observed effects on insulin. Gene expression data associated with Nrf-2 suggest, but do not confirm that the mechanism is related to changes in endogenous antioxidant defense, which is known to increase insulin sensitivity [42]. Collectively, the data suggest mango intake increased insulin sensitivity in individuals with chronic low-grade inflammation, possibly through activating the Nrf-2 gene and increasing cellular antioxidant status. The data support consuming mango fruit as part of a dietary pattern to address insulin resistance and warrant further research to understand the mechanisms underpinning the actions of mango intake.

## Figures and Tables

**Table 1:** Recipe and nutrient composition of study interventions *.

	Mango Intervention	Control Intervention
Serving size (g)	230	221.6
Kent mango (g)	126.9 g	0
Keitt mango (g)	38.1 g	0
Italian ice (g)	0	110
Crystal light mango flavor (g)	0	1.6
Water (g)	65	110
Total carbohydrate (g)	24.5	25
Total sugars (g)	22	25
Total protein (g)	1.5	0
Total fat (g)	0.75	0
Dietary fiber (g)	2.5	0
Total energy (kcal)	100	100

* Nutrients of food ingredients analyzed by Food Processor Pro SQL Edition by ESHA (version 10.15.41, ESHA Research, Salem, OR, USA).

**Table 2:** Demographic, metabolic, and health indices at week 0 visit.

Variables *	Control Intervention	Mango Intervention
Glucose (mg/dL)	117.68 ± 2.68	117.83 ± 1.98
Insulin (µIU/mL)	13.19 ± 2.55	12.42 ± 1.00
Total Cholesterol (mg/dL)	215.19 ± 10.35	213.74 ± 11.24
LDL Cholesterol (mg/dL)	139.04 ± 8.23	139.25 ± 7.77
HDL Cholesterol (mg/dL)	58.75 ± 4.02	53.38 ± 4.83
IL-6 (pg/mL)	1.84 ± 0.16	2.10 ± 0.28
TNFα (pg/mL)	0.99 ± 0.05	1.07 ± 0.08
hs-CRP (mg/L)	3.32 ± 0.56	3.01 ± 0.45
HOMA IR	3.99 ± 0.84	3.65 ± 0.33
Matsuda Index	4.07 ± 0.56	3.06 ± 0.31
Disposition Index	5.63 ± 1.43	3.64 ± 0.77
Insulinogenic Index	1.44 ± 0.29	1.16 ± 0.19
Glucose AUC_(0–120 min)_ (mg·min·dL^−1^)	19017.52 ± 1132.88	19643.50 ± 1168.21
Insulin AUC_(0–120 min)_ (µiU·min·mL^−1^)	7430.74 ± 958.79	8516.56 ± 908.84
BMI (kg/m^2^)	30.23 ± 0.95	30.72 ± 0.73
Age (years)	36.62 ± 2.86	38.75 ± 2.84
Caucasian:African American:Hispanic:Asian:Other (not known)	10:3:1:6:4	9:6:3:5:1
Male/Female	9/15	10/14

* Continuous variables are means and standard errors and were checked for differences between interventions.

**Table 3:** Body weight change in mango and control interventions.

Intervention	Body Weight at Week 0 (kg)	Body Weight at Week 4 (kg)	*p*-Value
Mango	84.35 ± 2.50	84.62 ± 2.56	0.37
Control	84.74 ± 3.11	85.61 ± 3.17	<0.001

Data are presented as means ± SEM.

**Table 4:** Metabolic assessments after mango and control interventions in participants with OW/OB and chronic low-grade inflammation.

Analyte/Marker	Mango Intervention Week 4	Control Intervention Week 4	*p*-Value
Glucose (mg/dL)	119.67 ± 1.02	116.95 ± 1.02	0.51
Insulin (µIU/mL)	8.2 ± 1.16	15.26 ± 1.18	0.05
Total Cholesterol (mg/dL)	202.21 ± 1.03	195.43 ± 1.03	0.46
LDL Cholesterol (mg/dL)	129.21 ± 1.03	125.60 ± 1.03	0.50
HDL Cholesterol (mg/dL)	47.33 ± 1.04	48.63 ± 1.04	0.61
Triglycerides (mg/dL)	97.16 ± 1.08	81.04 ± 1.08	0.09
HOMA-IR	2.28 ± 1.19	4.67 ± 1.21	0.03
Matsuda Index	3.85 ± 1.15	2.23 ± 1.17	0.07
Disposition Index	2.76 ± 1.02	5.37 ± 1.03	0.04
Insulinogenic Index	1.33 ± 1.22	0.81 ± 1.24	0.09
Glucose AUC_(0–120min)_ (mg min·dL^−1^)	16,634.13 ± 1.09	20,749.14 ± 1.10	0.18
Insulin AUC_(0–120min)_ (µiU·min·mL^−1^)	6137.62 ± 1.12	7261.06 ± 1.13	0.44

Values are least square means (LSMs) and standard error of the mean (SEM).

## Data Availability

The data presented in this study are available at the request from the corresponding author due to potential intellectual property.

## Abbreviations

T2DM	type 2 diabetes mellitus
OW/OB	overweight and obese
hs-CRP	high-sensitivity C-reactive protein
CVD	cardiovascular disease
NHANES	National Health and Nutrition Examination Survey
CNRC	Clinical Nutrition Research Center
CDC	Centers for Disease Control and Prevention
AHA	American Heart Association
OGTT	oral glucose tolerance test
EDTA	ethylenediaminetetraacetic acid
IL-6	interleukin 6
TNFα	tumor necrosis factor alpha
ELISA	enzyme-linked immunosorbent assay
cDNA	complementary DNA
qPCR	quantitative real-time polymerase chain reaction
TLR	Toll-like receptor
Nrf2	nuclear factor erythroid 2–related factor 2
GAPDH	glyceraldehyde-3-phosphate dehydrogenase
Ct	threshold cycle
RT	reverse transcription
HOMA-IR	Homeostasis Model Assessment of Insulin Resistance
IGI	insulinogenic index
DI	disposition index
AUCs	areas under the curves
BMI	body mass index
RM-ANOVA	repeated-measure of analysis of variance
ANCOVA	analysis of covariance
LSM	least square means
SEM	standard error of mean
PBMCs	peripheral blood mononuclear cells
NFкB	nuclear factor-kappa B transcription factor
QUICKI	quantitative insulin sensitivity check index
TAC	Total antioxidant capacity
LDL	low-density lipoprotein
HDL	high-density lipoprotein
